# Mutation-driven evolution of antibacterial function in an ancestral antifungal scaffold: Significance for peptide engineering

**DOI:** 10.3389/fmicb.2022.1053078

**Published:** 2022-12-01

**Authors:** Jing Gu, Noriyoshi Isozumi, Bin Gao, Shinya Ohki, Shunyi Zhu

**Affiliations:** ^1^Group of Peptide Biology and Evolution, State Key Laboratory of Integrated Management of Pest Insects and Rodents, Institute of Zoology, Chinese Academy of Sciences, Beijing, China; ^2^Center for Nano Materials and Technology (CNMT), Japan Advanced Institute of Science and Technology (JAIST), Nomi, Ishikawa, Japan

**Keywords:** drosomycin, mehamycin, nematode, co-option, allostery, deletion mutant, membrane permeability, functional diversification

## Abstract

Mutation-driven evolution of novel function on an old gene has been documented in many development- and adaptive immunity-related genes but is poorly understood in immune effector molecules. Drosomycin-type antifungal peptides (DTAFPs) are a family of defensin-type effectors found in plants and ecdysozoans. Their primitive function was to control fungal infection and then co-opted for fighting against bacterial infection in plants, insects, and nematodes. This provides a model to study the structural and evolutionary mechanisms behind such functional diversification. In the present study, we determined the solution structure of mehamycin, a DTAFP from the Northern root-knot nematode *Meloidogyne hapla* with antibacterial activity and an 18-mer insert, and studied the mutational effect through using a mutant with the insert deleted. Mehamycin adopts an expected cysteine-stabilized α-helix and β-sheet fold in its core scaffold and the inserted region, called single Disulfide Bridge-linked Domain (abbreviated as sDBD), forms an extended loop protruding from the scaffold. The latter folds into an amphipathic architecture stabilized by one disulfide bridge, which likely confers mehamycin a bacterial membrane permeability. Deletion of the sDBD remarkably decreased the ability but accompanying an increase in thermostability, indicative of a structure-function trade-off in the mehamycin evolution. Allosteric analysis revealed an interior interaction between the two domains, which might promote point mutations at some key sites of the core domain and ultimately give rise to the emergence of antibacterial function. Our work may be valuable in guiding protein engineering of mehamycin to improve its activity and stability.

## Introduction

Functional diversification (also known as functional shift) of genes primarily occurs among paralogs generated by gene duplications (Ganfornina and Sánchez, 1999; Philippe et al., 2003; Nei, 2013) but some studies have indicated that such event also occurs in orthologs formed by speciation (Ganfornina and Sánchez, 1999; Zhu et al., 2020). Antimicrobial peptides (AMPs), normally <100 amino acids in size, form a key component of an organism’s innate immune system, which show broad-spectrum antimicrobial activity against a range of pathogenic bacteria, fungi, viruses, and protozoa (Zasloff, 2002, 2019; Bulet et al., 2004). Because of their presence as either a single-copy or multi-copy form in different species and often exhibiting a differential antimicrobial spectrum, AMPs may become a model to study the evolutionary novelty of genes after gene duplication or speciation. A majority of the AMPs display hydrophobic and cationic properties, which allows them easily attach to and insert into membrane bilayers to damage the membrane structure. Also there exist non-membrane disruptive AMPs that target key cellular processes, such as DNA and protein synthesis, enzymatic activity and cell wall synthesis (Brogden, 2005; Nguyen et al., 2011). It is known that the AMPs sourced from vertebrates have developed their immunomodulatory capacity (Easton et al., 2009; Mansour et al., 2014), which likely interferes with the immune system functions when systematically administered (Zhu et al., 2022). However, some AMPs from the organisms evolutionarily distant from vertebrates could be viable drug candidates for dealing with the growing problem of antibiotic resistance (Fox, 2013; Magana et al., 2020; Zhu et al., 2022).

Defensins are a group of small cationic AMPs stabilized by several disulfide bridges, which can be classified as *cis* and *trans* based on their disulfide bridge connectivity patterns (Ganz, 2003; Silva et al., 2014; Shafee et al., 2016). The *cis*-defensins refer to a class of peptides with the cysteine-stabilized α-helix and β-sheet (CSαβ) motif (i.e., CSαβ-defensin) and they are distributed in invertebrates, plants, fungi and bacteria (Mygind et al., 2005; Zhu et al., 2005, 2020, 2022; Gao et al., 2009a; Shafee et al., 2016). The *trans*-defensins comprise α-, β-, and θ-defensins derived from vertebrates, and big defensins from invertebrates (Zhu and Gao, 2013; Shafee et al., 2016). Evolutionarily, both of them might originate from a common ancestry (Zhou et al., 2019). Based on the differences of sequences, origins, and functions, CSαβ-defensins can be further distinguished into three subgroups including antibacterial ancient invertebrate-type defensins (AITDs), antibacterial classical insect-type defensins (CITDs), and antifungal plant/insect-type defensins (PITDs) (Zhu, 2008).

**FIGURE 1:**
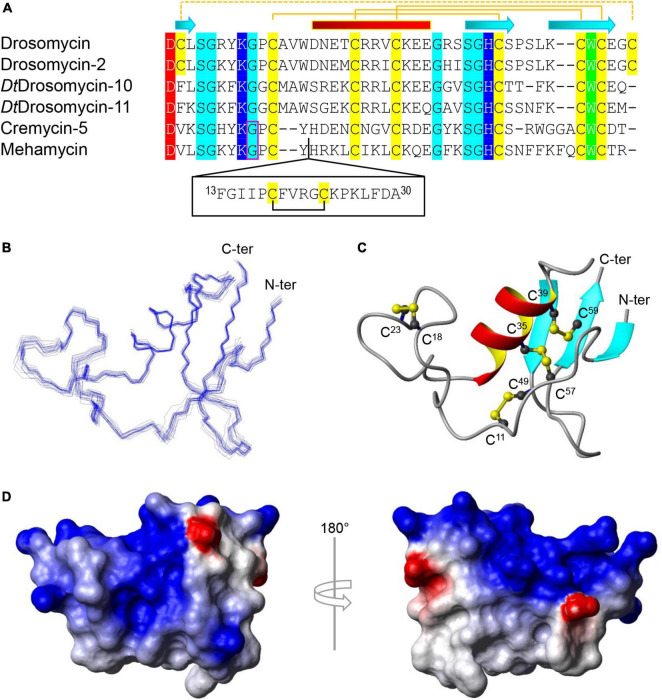
Sequence and structure of mehamycin. **(A)** Multiple sequence alignment (MSA) of mehamycin and other representative drosomycin-type antifungal peptides (DTAFPs) from *Drosophila* and nematodes. Identical acidic amino acids are shaded in red, basic in blue, hydrophobic in green, hydrophilic in cyan, and all cysteines in yellow. The pairing pattern of disulfide bridges and secondary structure elements (cylinder: α-helix; arrow: β-strand) are drawn according to the structural coordinates of drosomycin (pdb entry 1MNY). The dotted line indicates the disulfide bridge absent in other members. Phase-1 introns are boxed in pink. The proposed insertion of mehamycin is boxed in black. **(B–D)** Solution structure of mehamycin. **(B)** A family of 20 lowest energy structures superimposed over the backbone atoms of residues 1–61. **(C)** A ribbon model of a representative structure with disulfide connectivities shown in ball and stick model. The termini are labeled with N-ter and C-ter, and cysteines with their residue numbers. **(D)** Surface potential distribution with negatively charged, positively charged, and electrostatically neutral zones highlighted in red, blue, and white, respectively.

Drosomycin is the first inducible antifungal peptide originally isolated from the hemolymph of immune-challenged *Drosophila melanogaster*, which belongs to the member of the superfamily of CSαβ defensins (Fehlbaum et al., 1994; Landon et al., 1997; Zhang and Zhu, 2009). It is a small cationic peptide comprised of 44 residues stabilized by four disulfide bridges (Figure 1A), which selectively damages spores and hyphae of filamentous fungi through causing partial lysis (Fehlbaum et al., 1994; Landon et al., 1997; Gao and Zhu, 2008). Structure-function relationship studies of drosomycin have highlighted the functional role of seven charged and one aromatic residues in its antifungal activity (Zhang and Zhu, 2010; Zhu and Gao, 2014). Besides drosomycin, the *D. melanogaster* genome also encodes six additional paralogs (Drosomycin-1 to Drosomycin-6), all located on 3L chromosome arm with three distinct clusters, in which only Drosomycin-2 has been confirmed to have antifungal activity (Fehlbaum et al., 1994; Tian C. et al., 2008; Deng et al., 2009). Drosomycin-type antifungal peptides (DTAFPs) are restrictedly distributed in some ecdysozoans including three phyla (Arthropoda, Nematode, and Tardigrade), and nearly all species of plants (i.e., plant defensins), but absent in fungi and protozoans (Carvalho Ade and Gomes, 2011; Zhu and Gao, 2014). Such a patchy distribution pattern indicates a consequence of the plant-to-ecdysozoan horizontal gene transfer (Zhu and Gao, 2014).

The majority of DTAFPs exist as multigene families and some have evolved novel biological activities. For example, in *Drosophila takahashii*, two members (DtDrosomycin-11 and DtDrosomycin-11d) of the multigene family lost the disulfide bridge linking the amino- and carboxyl-termini of the peptide (Figure 1A), which leads to the evolutionary emergence of antibacterial activity accompanying the complete loss or a significant reduction in antifungal function (Gao and Zhu, 2016). In contrary to drosomycin from *Drosophila* that lack an intron, nematode DTAFPs have a conserved gene structure composed of two exons and one phase-1 intron (Figure 1A). Similarly, the multigene family of DTAFPs comprising 15 members (Cremycin-1 to Cremycin-15) in the fruit nematode *Caenorhabditis remanei* also lack the fourth disulfide bridge (Figure 1A). Consequently, Cremycin-5 exhibits activity against the yeast pathogen *Candida albicans* not limited to filamentous fungi, whereas Cremycin-15 has evolved an antibacterial activity (Zhu and Gao, 2014). It is also worth mentioning that DTAFPs in scorpions have been proposed to switch their targets to animal sodium channels (Cohen et al., 2009; Zhu et al., 2010, 2020). In addition to the typical DTAFPs in nematode species, there exists a class of structurally unique DTAFPs in the Northern root-knot nematode *Meloidogyne hapla* and the reniform nematode *Rotylenchulus reniformis*, which contain one insertion of 10–22 residues preceding the α-helix of the DTAFP scaffold, named single disulfide bridge-linked domain, abbreviated as sDBD (Zhu and Gao, 2014; Gu et al., 2018). Mehamycin derived from *M. hapla* is such a peptide with an 18-residue sDBD (Figure 1A), which is accompanied by functional change acquiring moderate antibacterial activity (Gu et al., 2018).

In this work, we report for the first time the experimental structure of mehamycin using the Nuclear Magnetic Resonance (NMR) Spectroscopy technique, and the comparison study on structure and function between mehamycin and its mutant with the sDBD deleted. Using allosteric analysis, we further revealed an interior interaction between its two domains, in which the insertion could promote the evolution of the core domain. This study will help us better understand the role of mutations in driving the functional novelty of a species-specific bi-functional defensin following speciation. In the meantime, it also provides new insights into evolution-guided design of peptide drugs.

## Materials and methods

### Recombinant expression vector construction

Inverse PCR was employed to generate mehamycin truncated mutant with the plasmid pET-28a-mehamycin previously constructed (Gu et al., 2018) as template. Two back-to-back primers were synthesized [Δ(F13-A30)-FP: CATCGTAAATTATGCATTAAGCTT; Δ(F13-A30)-RP: ATAACATGGACCTTTATATTTACC] by Beijing Genomics Institution (BGI)-Tech (Beijing, China). The amplification conditions were 5 min at 94°C followed by 30 cycles (45 s at 94°C, 45 s at 55°C, and 5 min at 72°C) with ExTaq DNA polymerase (Takara, Dalian, China). The PCR products were modified by 5′-end phosphorylation with T4 polynucleotide kinase (Takara) and ATP after end polishing with Pfu polymerase (CW Biotech, Beijing, China). Subsequently, the products circularized by T4 DNA ligase (Takara) were used for *Escherichia coli* DH5a transformation. Positive clones were confirmed by DNA sequencing (Tsingke Biological Technology, Beijing, China).

### Protein expression and purification

Recombinant plasmids were transformed into *E. coli* BL21 (DE3) pLysS cells grown in LB medium (1% tryptone, 0.5% yeast extract, and 0.5% NaCl, pH 7.2) for protein expression. The induction was initiated with 0.5 mM IPTG at an OD_600_ of 0.2. Cells were harvested after induction for 4 h at 37°C by centrifugation and were resuspended in buffer containing 0.1 M Tris–HCl (pH 8.5) and 0.1 M NaCl for sonication. *In vitro* refolding and purification of recombinant protein, expressed as inclusion body, was performed according to the previously reported method (Turkov et al., 1997; Zhu et al., 2013; Gu et al., 2018). Briefly, inclusion bodies were firstly washed with isolation solution containing 2 M urea and 2% Triton X-100, and then solubilized in denaturation buffer containing 6 M guanidine-HCl, 1 mM EDTA, 0.1 M Tris–HCl (pH 8.5), and 30 mM β-mercaptoethanol for 2 h. Refolding was performed by 20-fold dilution in 0.2 M ammonium acetate (pH 9.0) at room temperature for 48 h. Refolded protein was dissolved in water after salting out by 80% saturation of solid ammonium sulfate. The UV absorbance was monitored spectrophotometrically at a wavelength of 225 nm. The single well-defined peak of recombinant protein was collected and lyophilized by Thermo Scientific SAVANT SPD1010 SpeedVac Concentrator (USA). To produce ^15^N-labeled protein for NMR, *E. coli* cells transformed with the pET-28a-mehamycin were grown in M9 minimal medium (0.6% Na_2_HPO_4_, 0.3% KH_2_PO_4_, 0.05% NaCl, 0.1% ^15^NH_4_Cl, 0.2% glucose, 0.001% Thiamine, 0.012% MgSO_4_, 0.001% CaCl_2_, and 33 μM FeCl_3_). The labeled samples were prepared using the procedure described above. The purity and molecular mass was determined by matrix-assisted laser desorption/ionization time of flight mass spectrometry (MALDI-TOF MS) using an ultraflextreme instrument (Bruker, Rheinstetten, Germany) in the positive-ion reflection mode and a-cyano-4-hydroxycinnamic acid (CHCA) as a liquid matrix.

### Circular dichroism spectroscopy

Structural features of recombinant peptides were studied by circular dichroism (CD) spectroscopy analysis on Chirascan Plus spectropolarimeter v.4.4.0 (Applied Photophysics Ltd., UK) (Gao and Zhu, 2016). Spectra were recorded at 10–65°C with a quartz cell of 1.0 mm thickness with a peptide concentration of 0.1 mg/ml in water. The wavelengths used ranged from 185 to 260 nm. Data were collected at 1 nm intervals with a scan rate of 60 nm/min and expressed as delta epsilon (cm^–1^M^–1^) calculated as [θ × (MRW × 0.1)/(C × L)/3298], where θ is the ellipticity (in millidegrees), C is the concentration (in mg/ml), L is the pathlength (in cm), and MRW is the mean residue weight (in Da).

### Nuclear magnetic resonance spectroscopy

The purified ^15^N-labeled peptide was dissolved into H_2_O containing 10% D_2_O for NMR lock, and the pH was adjusted to 4.5 with DCl and NaOD. A set of the free induction decay (FID) data (^15^N-separated 3D-NOESY, ^15^N-separated 3D-TOCSY, ^1^H-^1^H 2D-NOESY and ^1^H-^1^H 2D-TOCSY) was recorded on a Bruker AVANCE-III 800. Mixing time of NOESY and spin lock time of TOCSY were set as 120 and 70 msec, respectively. During the NMR experiments, the sample temperature was kept at 25°C. All FID data were processed and displayed with NMRPipe (Delaglio et al., 1995). The NMR spectra were analyzed with Sparky.^1^ The three-dimensional structure was elucidated with CYANA-2.1 (Lopez-Mendez and Güntert, 2006) and Xplor-NIH (Schwieters et al., 2003). Structural figures were generated with MOLMOL (Koradi et al., 1996). The chemical shift data and coordinates of mehamycin were deposited to BioMagResBank (accession number 36511) and ProteinDataBank (accession number 8GXT), respectively.

### Antimicrobial activity assay

Lethal concentration (C*_*L*_*) of a peptide was determined by inhibition zone assay performed according to the previous procedure (Hultmark, 1998; Ekengren and Hultmark, 1999). Briefly, filamentous fungi were incubated on potato dextrose agar (PDA) (20% potato, 2% glucose, and 1.5% agar) plate at 30°C for 1 week. Spores were harvested and suspended in sterile water with an OD_600_ of 0.5. A total of 6-ml preheated PDA containing 0.8% agar was mixed with 50 μl spores suspension and poured into 9-cm Petri dishes, giving a depth of 1 mm. 2-mm wells were punched in the plate and then filled with 2 μl of two-fold serially diluted peptides at three different doses. The agar plates were incubated overnight at 30°C, and zones of inhibition were measured. Bacteria or *C. albicans* respectively grown in LB medium or potato dextrose broth (PDB) (20% potato and 2% glucose) at an OD_600_ of 0.5 were used as the same procedures described above. A lethal concentration (C*_*L*_*) was calculated from a plot of d^2^ against log n, where d is the diameter (in cm) of inhibition zone and *n* is the amount of peptide applied in the well (in nmol). The plot is linear and thus C*_*L*_* can be calculated from the slope (k) and the intercept (m) of this plot. The formula used here is C*_*L*_* = 2.93/ak10^m/k^, where a is the agar depth (in cm) and C*_*L*_* is in μM. Sources of microbial strains used in this assay are provided in Supplementary Table 1.

### Membrane permeability assay

The membrane permeability assay was performed according to the previous procedure (Zhu et al., 2022). 5 × 10^5^ *Bacillus megaterium* cells in 500 μL of phosphate buffered saline (PBS) (pH 7.3) were incubated with 1 μM propidium iodide (PI) for 5 min in the dark. Fluorescence was measured using the F-7000 spectrophotometer (Hitachi High-Technology Company, Japan). Once basal fluorescence reached a constant value, peptides or vancomycin at 2.5 or 5 × C*_*L*_* were added, and changes in fluorescence arbitrary were monitored (λ_exc_ = 525 nm; λ_ems_ = 595 nm). Vancomycin, a bacterial cell-wall synthesis inhibitor without cellular permeability, and Meucin-18, a scorpion venom-derived lytic peptide (Gao et al., 2009b), were used as negative and positive control, respectively.

### Residue relevance analysis

ProteinLens (Mersmann et al., 2021), an atomistic graph-theoretical method for the investigation of allosteric signaling within one molecule, was used to analyze potential residue relevance between sDBD and drosomycin-like domain (DLD) subdomains of mehamycin. Three cationic residues derived from the inserted domain were selected as source sites given their potential functional importance. The server used was at https://www.proteinlens.io/webserver/.

## Results

### Mehamycin adopts a cysteine-stabilized α-helix and β-sheet fold with a disulfide bridge-stabilized extended loop

**FIGURE 2:**
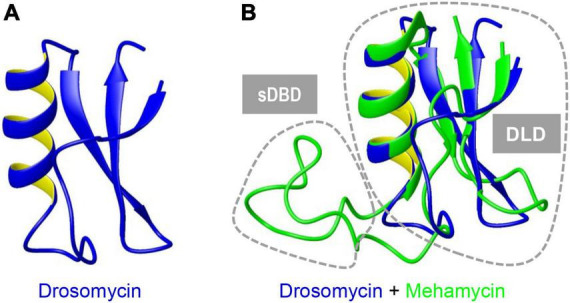
Superimposition of mehamycin and drosomycin revealing their high structural similarity. Drosomycin **(A)**. Mehamycin and drosomycin **(B)**. Two distinct structural subdomains [single Disulfide Bridge-linked Domain (sDBD) and drosomycin-like domain, abbreviated as DLD] are indicated by dotted circles.

To investigate the structure and function relationship of mehamycin, we firstly determined its experimental structure using NMR spectroscopy analysis of the ^15^N-labeled protein (Figure 1B). NMR-derived constraints and structural statistics are summarized in Table 1. Only 28% residues in the molecule form the regular secondary structure, thus large part of mehamycin exists as random coil. Because of such structural property, number of observed nuclear Overhauser effects (NOEs) is relatively small, including 690 signals (325 for Short-range, 156 for Medium range, and 209 for Long range), and less than that we expected for the molecular size. Therefore, the solution NMR structures were calculated with limited structural restraints and the final 20 structures showed moderate score in Ramachandran analysis with PROCHECK, with 51% residues in favored regions, 30% in additionally allowed regions, 12% in generously allowed regions, and 3.5% in disallowed regions. For the final 20 structures, no violations were found in distance (>0.5 Å) and angle (>5 degrees) restraints. The resulting family of 20 structures is shown in Figure 1B, and the ribbon model and the molecular surface colored with different charge distributions in Figures 1C,D, respectively. Mehamycin adopts a typical cysteine-stabilized α-helix and β-sheet (CSαβ) fold in its core scaffold, comprising an α-helix and a three-stranded antiparallel β-sheet stabilized by three disulfide bridges (Cys11–Cys49, Cys35–Cys57, and Cys39–Cys59). In the mehamycin structure, the helix region spans from residue 31 to 39, and the three β-strands are formed by residues 2–3, 46–48, and 58–60. Its core scaffold is highly similar to that of drosomycin with a root mean square deviation (RMSD) of 1.38 Å calculated from 35 structurally equivalent residues (Figure 2). Different from the previously computationally predicted structure, the sDBD forms an extended loop protruding from the scaffold stabilized by one disulfide bridge (Cys18–Cys23) other than a predicted α-helical conformation (Gu et al., 2018).

**TABLE 1:** Nuclear magnetic resonance (NMR)-derived constraints and structural statistics of the final 20 coordinates.

Number of experimental restraints	
Total number of NOEs	690
Short-range | i–j | ≤1	325
Medium range 1< | i–j | <5	156
Long range 5≤ | i–j |	209
Hydrogen bond restraints	22
Dihedral angle restrains (phi, psi)	18, 18
**Number of violations**	
NOEs (>0.5 Å)	0
Dihedral angles (>0.5 deg.)	0
**Average pairwise r. m. s. d. (Å)**	
Backbone atoms (residues 2–60)	0.42 ± 0.10
Heavy atoms (residues 2–60)	1.26 ± 0.14
Backbone atoms of ordered residues	0.16 ± 0.04
Heavy atoms of ordered residues	0.98 ± 0.26
**Ramachandran statistics (%)**	
Residues in favored regions	50.6 ± 1.9
Residues in additionally allowed regions	30.0 ± 3.0
Residues in generously allowed regions	12.2 ± 2.6
Residues in disallowed regions	3.5 ± 0.9

### Identification of mehamycin truncated mutant

**FIGURE 3:**
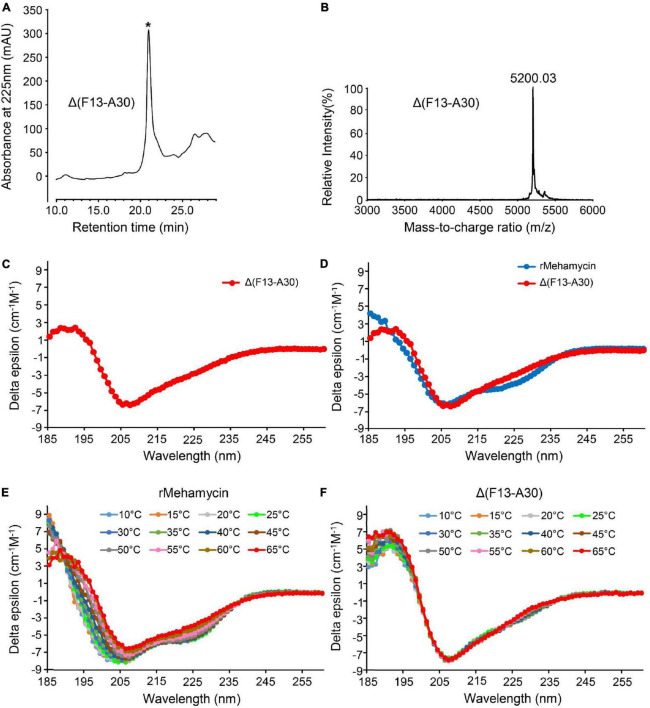
Characterization of mehamycin-del (F13-A30). **(A)** Reverse phase high-performance liquid chromatography (RP-HPLC) showing the retention time (TR) of Δ(F13-A30), indicated by an asterisk. **(B)** MALDI-TOF MS of HPLC-purified Δ(F13-A30). **(C)** CD spectra of Δ(F13-A30) at room temperature. **(D)** CD spectral superimposition of mehamycin and Δ(F13-A30) at room temperature. **(E,F)** CD spectra of mehamycin and Δ(F13-A30), measured at 10–65°C.

To explore the effect of the insertion on the structural and functional features of mehamycin, we used inverse PCR mutagenesis to delete the insertion (Phe13-Ala30) and prepared the recombinant truncated mutant named Δ(F13-A30) (Supplementary Figures 1, 2). Δ(F13-A30) was obtained from *E. coli* inclusion bodies with only one extra N-terminal Met, and thus *in vitro* refolding was carried out. The product was further purified by reverse phase high-performance liquid chromatography (RP-HPLC) with a retention time of 21 min as a totally symmetrical peak on the C18 analytical column (Figure 3A). The expression level was about 300 μg/L bacterial culture. The experimental average molecular mass was 5,200.03 Da determined by MALDI-TOF (Figure 3B), well matching the theoretical value of Δ(F13-A30) with an extra Met (5,201.19 Da) calculated from the sequence with six hydrogens removed due to the presence of three disulfide bridges. Using CD spectroscopy analysis, we evaluated the secondary structure of Δ(F13-A30), and confirmed its CSαβ structure, as identified by a positive maximum at 191 nm and a negative minimum at 207 nm (Figure 3C). In comparison with mehamycin, Δ(F13-A30) lacked a negative minimum around at 217–218 nm, a signature of α-helix, indicating that removal of the insertion decreased its helical content (Figure 3D).

### Δ(F13-A30) showing enhanced thermostability than the wild-type peptide

To investigate whether the insertion has an impact on the thermostability of mehamycin, we compared the temperature-induced structural changes between mehamycin and its truncated mutant Δ(F13-A30) using CD spectroscopy analysis (Figures 3E,F). With the temperature rise, the CD spectra of mehamycin underwent obvious right-shift accompanied with a decrease of negative ellipticity around 206 nm and a loss of negative band at 217–218 nm. Furthermore, the spectra showed significant changes at 185–190 nm measured beyond 55°C, indicative of its declined structural stability. In comparison with mehamycin, the spectra of Δ(F13-A30) displayed less alteration even when the temperature increased to 65°C. Taken together, our data demonstrated that the removal of sDBD can enhance the thermostability of mehamycin, indicative of a structure-function trade-off in the mehamycin evolution, as observed in the evolution of some enzymes, in which they obtained new enzymatic specificities but accompanied the loss of the protein’s stability (Shoichet et al., 1995; Tokuriki et al., 2008).

### Δ(F13-A30) showing some different antimicrobial activity from the wild-type peptide

Using the classical inhibition-zone assay, we quantitatively evaluated the antimicrobial activity of Δ(F13-A30) and compared it with that of mehamycin previously reported (Gu et al., 2018). The results are summarized as follows (Table 2): (1) Δ(F13-A30) showed weaker antifungal activity than mehamycin against three fungi tested here (*Neurospora crassa*, *Geotrichum candidum*, and *C. albicans*). (2) In comparison with mehamycin that had moderate antibacterial activity, Δ(F13-A30) showed a relatively higher activity on an array of Gram-positive bacteria except *B. subtilis*, with a lethal concentration (C*_*L*_*) ranging from 2.08 to 3.86 μM. For example, for three oral *Streptococcus* bacteria (*Streptococcus mutans*, *Streptococcus salivarius*, and *Streptococcus sanguinis*) (Zhu et al., 2022), the C*_*L*_* ranged from 2.08 to 2.93 μM; For the endospore forming bacterium *B. megaterium* and a penicillin-resistant clinical isolate (*Staphylococcus aureus* P1383), the C*_*L*_* determined were respectively 2.69 and 3.86 μM. (3) Consistently to mehamycin, Δ(F13-A30) also exhibited no activity on *E. coli* at the concentration range used here. Such an opposite change in the antimicrobial activity after the deletion of sDBD likely reflects a difference in their action modes of fungal and bacterial killing.

**TABLE 2:** Comparison of lethal concentrations (C*_*L*_*) of Δ(F13-A30) and mehamycin.

Microorganism	C*_*L*_*(μM)	
	Δ(F13-A30)	Mehamycin*
**Fungi**		
*Neurospora crassa* CGMCC 3.1605	33.78	26.01
*Geotrichum candidum* CCTCC AY 93038	53.56	35.05
*Candida albicans* 2.4116	77.98	29.64
**Gram-positive bacteria**		
*Bacillus megaterium* CGMCC 1.0459	2.69	8.97
*Bacillus subtilis* CGMCC 1.2428	14.49	10.91
*Micrococcus luteus* CGMCC 1.0290	3.11	8.67
Penicillin-resistant *Staphylococcus aureus* P1383	3.86	8.07
*Streptococcus mutans* CGMCC 1.2499 (ATCC 25175)	2.08	8.45
*Streptococcus salivarius* CGMCC 1.2498 (ATCC 7073)	2.26	8.93
*Streptococcus sanguinis* CGMCC 1.2497 (ATCC 49295)	2.93	5.16
**Gram-negative bacteria**	N.A.	N.A.
*Escherichia coli* ATCC 25922		

N.A., no activity, indicating no inhibition zone observed at 1.0 nmol peptide per well.

*Data derived from the reference.

### The role of single disulfide bridge-linked domain in conferring the membrane-disruptive ability of mehamycin

**FIGURE 4:**
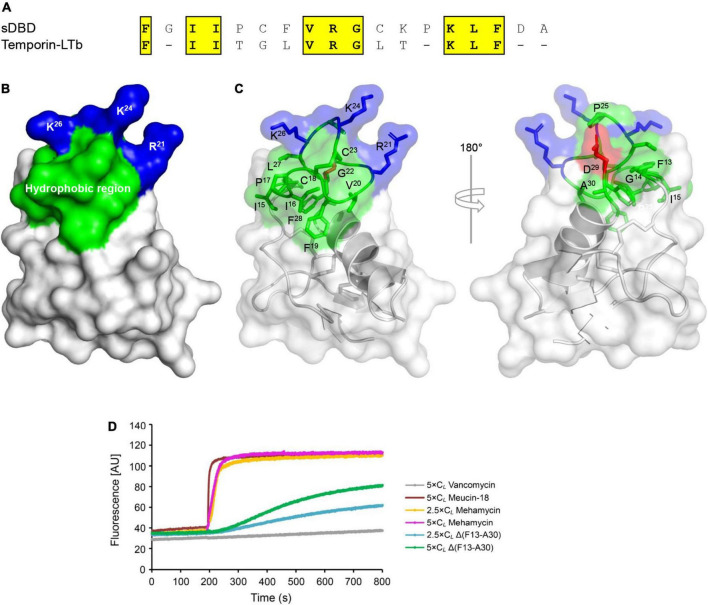
Sequence and structural bases of the membrane-disruptive ability of mehamycin. **(A)** Alignment of sDBD with a representative cationic lytic amphipathic peptide (Temporin–LTb) from frog skin. Identical amino acids are shaded in yellow. **(B,C)** The sDBD presents a typical local amphipathic architecture. The structure is shown as cartoons in a transparency mode in which residues involved in amphipathy are shown as sticks and colored in blue (basic), green (hydrophobic), and red (acidic), respectively. **(D)** Comparison of bacterial membrane permeation ability between mehamycin and Δ(F13-A30). Mehamycin and Δ(F13-A30) at 2.5 × or 5 × C*_*L*_* was added when the basal fluorescence remained constant for 200 s. Vancomycin (C*_*L*_*: 0.119 μM) and Meucin-18 (C*_*L*_*: 0.25 μM) were used as negative and positive control.

Compared with the primitive antifungal function, mehamycin has evolved a new antibacterial function, which is presumably originated from the insertion. To provide evidence in support of our opinion, we firstly carried out a survey of the AMP database^2^ and found that the sDBD shared about 30–50% sequence identity to a series of short, cationic AMPs essentially with a helical amphipathic design (Figure 4A). Subsequently, we analyzed the structural organization of the sDBD in the mehamycin structure and found that although this domain adopts an extended loop conformation, the disulfide bridge seems to provide some stabilized force to reduce its structural flexibility, allowing the formation of an amphipathic architecture (Figures 4B,C). In this design, three cationic residues (R^21^, K^24^, and K^26^) and a cluster of hydrophobic residues are segregated spatially into two distinct subdomains (Figures 4B,C).

Based on these observations, we examined the membrane permeability of mehamycin on *B. megaterium* using propidium iodide (PI), a fluorescent nucleic acid-binding dye, and compared it with that of Δ(F13-A30). Vancomycin (an antibiotic inhibitor of bacterial cell wall synthesis) and Meucin-18 (a pore-forming peptide from scorpion venom) (Gao et al., 2009b) were used as negative and positive controls, respectively. As shown in Figure 4D, mehamycin at 2.5 × C*_*L*_* and 5 × C*_*L*_* caused an immediate fluorescence increase as observed in the cells treated by Meucin-18, indicating that the bacterial membrane integrity was destroyed. In striking contrast to the parent peptide, Δ(F13-A30) only showed a much more subdued fluorescence rise in the *B. megaterium* cells (Figure 4D). This experiment revealed the role of the insertion in conferring the membrane permeability of mehamycin through its amphipathic design.

### Evidence for allosteric interaction between the single disulfide bridge-linked domain and drosomycin-like domain

**FIGURE 5:**
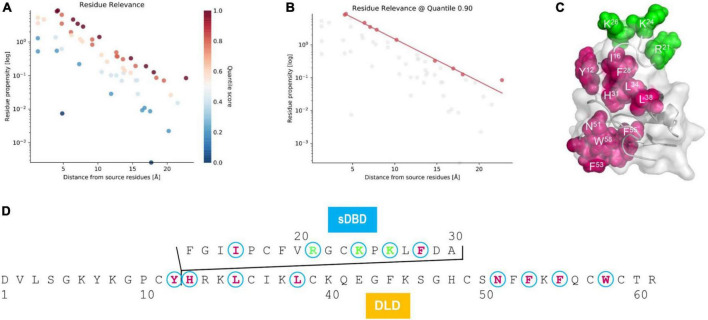
Residue relevance between sDBD and DLD subdomains of mehamycin. **(A)** The hotspot view of high or low connectivity to the source sites (R^21^, K^24^, and K^26^). All data points were plotted as propensity over distance from source, in which hotspots and coldspots are colored according to their quantile scores. **(B)** The relevant residues view at 0.90 quantile score cut off. **(C)** Mapping of relevant residues (red) to the source sites (green) on the structure of mehamycin. **(D)** Mapping of relevant residues (red) to the source sites (green) on the sequence of mehamycin.

As mentioned previously, the presence of sDBD is a unique trait that was only found in two nematode species. To investigate the effect of this functional domain on the evolution of the ancestral scaffold, we employed an allosteric analysis method, ProteinLens, to predict the potential residue relevance within mehamycin. By using the three cationic residues (R^21^, K^24^, and K^26^) located on the sDBD as source, we identified ten sites which are likely associated with the source residues (Figures 5A,B), of which eight are derived from the DLD domain (Figures 5C,D), including Y^12^, H^31^, L^34^, L^38^, N^51^, F^53^, F^55^, and W^58^, in support of the presence of allosteric communication between them. Of these ten relevant residues, seven (I^16^, F^28^, L^34^, L^38^, F^53^, F^55^, and W^58^) are hydrophobic and two of them (L^34^ and F^55^) are mehamycin-specific when compared with its ortholog Cremycin-5. In the latter, the equivalent residues are N and G, respectively (Figures 1, 5D). This observation suggests that this allostery could be mediated by a hydrophobic pathway to transfer the signal from the sDBD to DLD. This kind of interactions between two domains and the hydrophobicity increase in the whole molecule could commonly contribute the emergence of antibacterial function in mehamycin *via* disrupting the bacterial membrane structure.

## Discussion

Nematodes are one of the largest animal phylum, which are distributed in virtually all terrestrial and aquatic habitats. To confront microbe-rich environments during at least part of life cycle, nematodes have evolved many kinds of AMPs as the first line of defense against microbial infection, including antibacterial factors (ABFs), nemapores, cecropins, and caenacins/neuropeptide-like protein (Tarr, 2012; Midha et al., 2017). Additionally, a minor multiple gene family of DTAFPs were identified in *C. remanei*, in which Cremycin-5 displays strict fungicidal activity against filamentous fungi and several clinical isolates of *C. albicans* (Zhu and Gao, 2014). In *Caenorhabditis latens*, Clatencin-5, the ortholog of Cremycin-5 with two substitutions, also exhibits strong antifungal activity (Gu et al., 2021). Mehamycin is a bi-functional nematode AMP with two interacting domains. In view of its unique structural and functional features, more detailed characterization of other nematode-derived DTAFPs should be encouraged since this will help provide new clues for exploring new-type peptide antibiotics.

This study reports for the first time the NMR structure of mehamycin. The 18-residues insertion preceding α-helix of mehamycin does not change its global CSαβ structure and forms an extended loop connected by an extra disulfide bridge. Indeed, the CSαβ structural motif has the potential to tolerate insertions, deletions and substitutions throughout the structure, thus considered as a candidate for engineering design (Zhu et al., 2005). For example, MeuNaTxα-3, an α-scorpion toxin from *Mesobuthus eupeus*, possesses a J-loop insertion, which has been explored as a protein scaffold to graft exogenous antiviral and antibacterial motif (Zhu et al., 2012; Zhang et al., 2016). Therefore, the development of mehamycin-based scaffold deserves further study and exploration.

The truncated mutant shows higher structural stability and weaker membrane permeability compared with mehamycin, suggesting that the insertion plays a key role in bactericidal mechanism likely through structural adjustment to form a local amphipathic architecture. In addition, the gain of the insertion can be considered as an evolutionary advantage to *M. hapla* through expanding the antimicrobial spectrum and accelerating to kill pathogens. By analyzing sequence and structural features of the insertion, the sDBD in mehamycin shows high sequence identity with a cationic lytic amphipathic peptide (Temporin-LTb) from frog skin, and presents a typical local amphipathic architecture with segregated cationic and hydrophobic residues. This could promote electrostatic and hydrophobic interactions of mehamycin with anionic bacterial membrane, leading to an increased membrane-disruptive ability. It is worth noting that the deletion of sDBD in mehamycin led to an increase in the antibacterial activity. This might be a consequence of the removal of an acidic residue (D^29^) by this deletion since it has been found that in some defensins (e.g., micasin and Cremycin-5), an acidic residue often acts as a trade-off residue to maintain protein homeostasis (Wu et al., 2016; Gu et al., 2021). This can be further verified by saturation mutagenesis of D^29^ in mehamycin. Further measurements of antimicrobial activity against microbes from ecological niche of nematodes are needed to fully understand the biological and evolutionary significance of the insertion.

**FIGURE 6:**
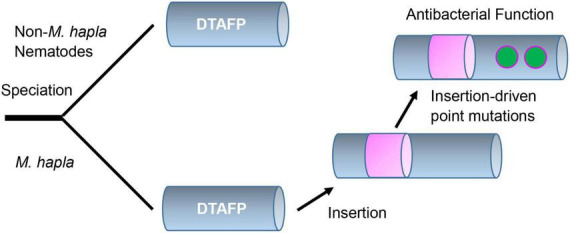
Proposed evolutionary history of mehamycin. In this evolutionary scenario, the ancient drosomycin-type antifungal peptide (DTAFP) gained an insert after speciation from other nematodes, which further drove point mutations at key sites located on its flanking core scaffold region and ultimately led to the emergence of antibacterial function. The insert is represented by a pink cylinder and point mutations are represented by green dots.

Taken together, our structural and functional data suggest that the evolution of new function in mehamycin is driven by an insertion event (Figure 6). Different from drosomycin and Cremycin-5 that display strict antifungal activity, the DLD in mehamycin alone possesses both antifungal and antibacterial activities, suggesting that point mutations have occurred in this domain. Because prior studies have shown that in eukaryotic genomes indel mutations often induce an increase in the substitution rate of their flanking regions (Tian D. et al., 2008; Zhang et al., 2011), we proposed that the sDBD could further drive point mutations at key sites of the core domain to form a functional pathway to mediate two-domain communication and the emergence of antibacterial function (Figure 6). In addition, the finding of the allosteric residues could be useful as candidates for peptide engineering to improve the activity and stability of mehamycin based on their tight connection with the proposed functionally important cationic residues involved in membrane disruption.

## Data availability statement

The data underlying are available in this article and in its online Supplementary material.

## Author contributions

SZ conceived and designed the study. JG, NI, BG, and SO performed the experiments. JG and SZ commonly wrote the manuscript with assistance from all other authors. All authors contributed to the article and approved the submitted version.

## Footnotes

1
http://www.cgl.ucsf.edu/home/sparky

2
https://aps.unmc.edu/
