# Supplementary material for: Mutation-driven evolution of antibacterial function in an ancestral antifungal scaffold: Significance for peptide engineering
Source: Front Microbiol. 2022 Dec 1;13:1053078. doi: 10.3389/fmicb.2022.1053078 (PMC9751787; doi:10.3389/fmicb.2022.1053078)
Supplement: Supplementary file 1 [file Data_Sheet_1.docx]

Supplementary Material

**Mutation-Driven Evolution of Antibacterial Function in an Ancestral Antifungal Scaffold: Significance for Peptide Engineering**

**Jing Gu^1^, Noriyoshi Isozumi^2^, Bin Gao^1^, Shinya Ohki^2^, and Shunyi Zhu^1*^**

^1^Group of Peptide Biology and Evolution, State Key Laboratory of Integrated Management of Pest Insects and Rodents, Institute of Zoology, Chinese Academy of Sciences, 1 Beichen West Road, Chaoyang District, Beijing 100101, China

^2^Center for Nano Materials and Technology (CNMT), Japan Advanced Institute of Science and Technology (JAIST), 1-1 Asahidai, Nomi, Ishikawa 923-1292, Japan

*** Correspondence:**Shunyi Zhu
Zhusy@ioz.ac.cn


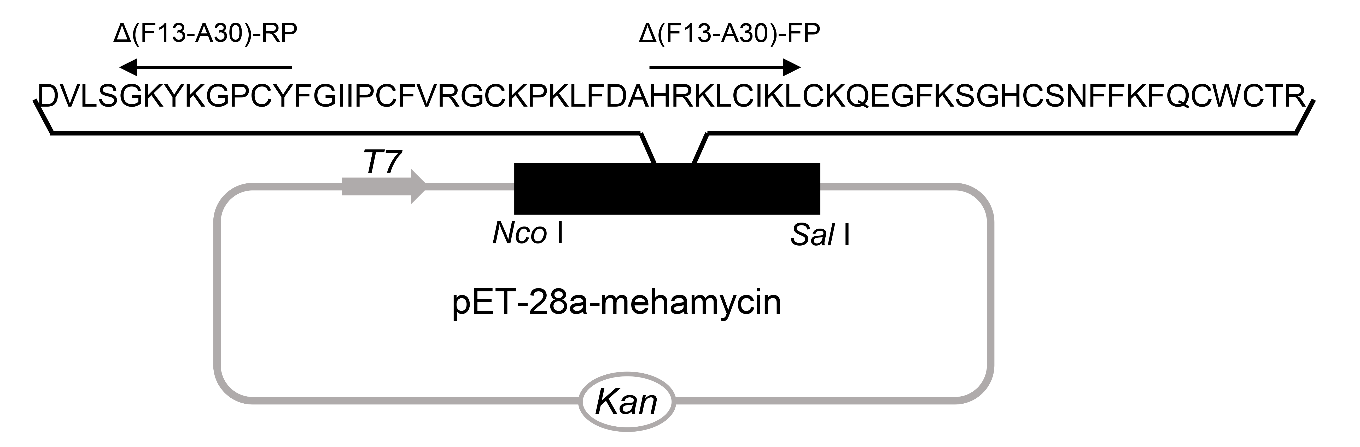


mehamycin

**A**

**B**


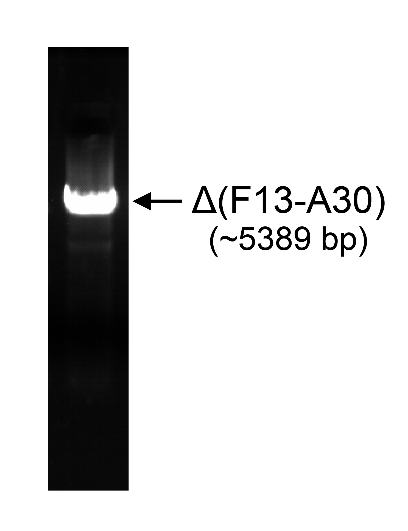


**Figure S1. Construction of pET-28a-mehamycin-Δ(F13-A30) recombinant expression vector.** (A) The inverse PCR strategy employed to construct the vector. The plasmid pET-28a-mehamycin was used as template for PCR amplification by two back-to-back primers (Δ(F13-A30)-FP and Δ(F13-A30)-RP) labeled by arrows. (B) The amplified PCR product.


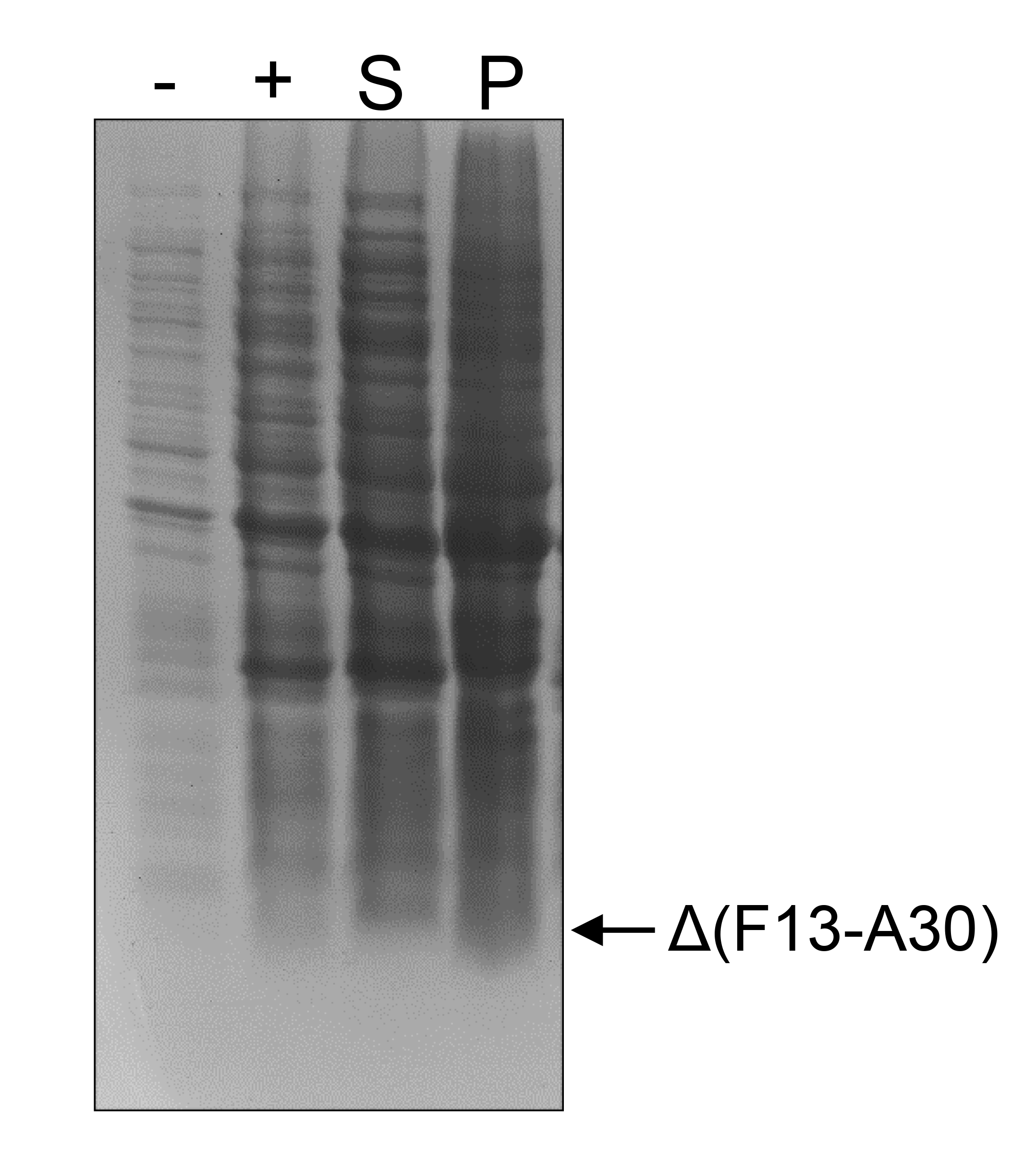


**Figure S2. SDS-PAGE showing the expression of mehamycin truncated mutant Δ(F13-A30) in *E. coli* BL21(DE3) pLysS cells.** - or +: total extract of *E. coli* cells carrying the Δ(F13-A30) plasmid without or with IPTG; S and P respectively represent supernatant and pellet prepared from the cell lysate by sonication; The recombinant peptide is labeled by arrow.

**Table S1. Microorganisms used in this study**

| Microorganism | Source |
| --- | --- |
| **Fungi** |  |
| Neurospora crassa CGMCC 3.1605 | Center for Microbial Resources, Institute of Microbiology, Beijing, China |
| *Geotrichum candidum* CCTCC AY 93038 | China Center for Type Culture Collection, Wuhan University, Wuhan, China |
| *Candida albicans* 2.4116 | Prof. Fengyan Bai, Institute of Microbiology, Beijing, China |
| **Gram-positive bacteria** |  |
| *Bacillus megaterium* CGMCC 1.0459 | Center for Microbial Resources, Institute of Microbiology, Beijing, China |
| *Bacillus subtilis* CGMCC 1.2428 | Center for Microbial Resources, Institute of Microbiology, Beijing, China |
| *Micrococcus luteus* CGMCC 1.0290 | Center for Microbial Resources, Institute of Microbiology, Beijing, China |
| Penicillin-resistant *Staphylococcus aureus* P1383 | 302nd Military Hospital, Beijing, China |
| *Streptococcus mutans* CGMCC 1.2499 (ATCC 25175) | Center for Microbial Resources, Institute of Microbiology, Beijing, China |
| *Streptococcus salivarius* CGMCC 1.2498 (ATCC 7073) | Center for Microbial Resources, Institute of Microbiology, Beijing, China |
| *Streptococcus sanguinis* CGMCC 1.2497 (ATCC 49295) | Center for Microbial Resources, Institute of Microbiology, Beijing, China |
| **Gram-negative bacteria** |  |
| *Escherichia coli* ATCC 25922 | Center for Microbial Resources, Institute of Microbiology, Beijing, China |
